# A Multifactorial Evaluation of the Effects of Air Pollution and Meteorological Factors on Asthma Exacerbation

**DOI:** 10.3390/ijerph17114010

**Published:** 2020-06-04

**Authors:** Hong-Ren Yu, Chun-Hung Richard Lin, Jui-Hsiu Tsai, Yun-Ting Hsieh, Ti-An Tsai, Chang-Ku Tsai, Yi-Chen Lee, Ta-Yu Liu, Chih-Min Tsai, Chih-Cheng Chen, Chih-Hao Chang, Te-Yao Hsu, Chen-Kuang Niu

**Affiliations:** 1 Department of Pediatrics, Chang Gung Memorial Hospital-Kaohsiung Medical Center, Kaohsiung 83301, Taiwan; yuu2004taiwan@yahoo.com.tw (H.-R.Y.); tiantsai@cgmh.org.tw (T.-A.T.); wind518@cgmh.org.tw (C.-K.T.); eugenelee20@gmail.com (Y.-C.L.); bigball@cgmh.org.tw (T.-Y.L.); tcmnor@adm.cgmh.org.tw (C.-M.T.); charllysc@adm.cgmh.org.tw (C.-C.C.); niubebe.clinic@msa.hinet.net (C.-K.N.); 2 Graduate Institute of Clinical Medical Sciences, College of Medicine, Chang Gung University, Kaohsiung 83301, Taiwan; 3 Department of Computer Science and Engineering, National Sun Yat-sen University, Kaohsiung 80424, Taiwan; d7837295@gmail.com; 4 Department of Psychiatry, Dalin Tzu Chi Hospital, Buddhist Tzu Chi Medical Foundation, Chia-Yi 62247, Taiwan; faanvangogh@gmail.com; 5 PhD Program in Environmental and Occupation Medicine, (Taiwan) National Health Research Institutes and Kaohsiung Medical University, Kaohsiung 80737, Taiwan; 6 Department of Respiratory Therapy, Chang Gung Memorial Hospital–Kaohsiung Medical Center, Kaohsiung 83301, Taiwan; windykevin@cgmh.org.tw; 7 Department of Obstetrics, Chang Gung Memorial Hospital-Kaohsiung Medical Center, Kaohsiung 83301, Taiwan; tyhsu@adm.cgmh.org.tw

**Keywords:** air pollution, meteorological factors, asthma, exacerbation, emergency room

## Abstract

In the real world, dynamic changes in air pollutants and meteorological factors coexist simultaneously. Studies identifying the effects of individual pollutants on acute exacerbation (AE) of asthma may overlook the health effects of the overall combination. A comprehensive study examining the influence of air pollution and meteorological factors is required. Asthma AE data from emergency room visits were collected from the Taiwan National Health Insurance Research Database. Complete monitoring data for air pollutants (SO_2_; NO_2_; O_3_; CO; PM_2.5_; PM_10_) and meteorological factors were collected from the Environmental Protection Agency monitoring stations. A bi-directional case-crossover analysis was used to investigate the effects of air pollution and meteorological factors on asthma AE. Among age group divisions, a 1 °C temperature increase was a protective factor for asthma ER visits with OR = 0.981 (95% CI, 0.971–0.991) and 0.985 (95% CI, 0.975–0.994) for pediatric and adult patients, respectively. Children, especially younger females, are more susceptible to asthma AE due to the effects of outdoor air pollution than adults. Meteorological factors are important modulators for asthma AE in both asthmatic children and adults. When studying the effects of air pollution on asthma AE, meteorological factors should be considered.

## 1. Introduction

According to the 2010 Global Disease Burden Assessment, outdoor air pollution caused more than three percent of the annual disability and life lost. This is a pronounced increase from the previous report [1]. Urbanization is an important cause for the increase in asthma, and this may be partly attributed to increased outdoor air pollution [2,3]. In a study of ten European cities, 14% of pediatric asthma occurrences and 15% of pediatric asthma acute exacerbations (AE) were associated with traffic-related contaminants [4]. Air pollution promotes the occurrence and progression of asthma through several mechanisms, including tissue damage by oxidative stress, inflammation and immune response, increased airway responsiveness, and airway remodeling [5,6]. Particulate matter (PM) often contains many immunogenic substances, such as fungal spores and pollen, which are linked to the worsening of asthmatic symptoms [7,8]. Ozone (O_3_) exposure has been proven to induce airway inflammation and airway hyperresponsiveness, leading to lung function impairment and asthma attacks [9,10,11]. Sulfur dioxide (SO_2_) can induce bronchospasms and asthma AE in asthmatic patients [12]. High exposure to nitrogen dioxide (NO_2_) is associated with a reduced bronchodilator effect, worsened lung function, and symptomatic exacerbations of asthma [13,14,15]. 

In Taiwan, Wang et al. showed that exposure to particulate matter with an aerodynamic diameter of less than 10 μm (PM_10_), particles with an aerodynamic diameter of less than 2.5 μm (PM_2.5_), and carbon monoxide (CO) was associated with the risk of asthma in kindergarten children [16]. PM_2.5_ had a synergistic effect in conjunction with dust mites for the development of asthma. While the asthma prevalence rates in children and adults were about 7.5% and 11.5%, respectively, in 2011, and showed an upward trend [17,18], there was a downward trend for PM_2.5_ and other certain air pollutants in this time period [19]. Thus, single air pollutant effects cannot adequately explain this observation.

In addition to the effects of pollutants on asthma [10,11,12,13,14,15], another area among the numerous factors affecting asthma occurrence is the meteorological factor. Previous studies have provided the effects of several meteorological factors on asthma acute attacks, such as temperature change, high atmospheric pressure, low relative humidity, and substantial changes in humidity [20,21,22,23,24]. However, in the real world, the dynamic change in air pollutants and meteorological factors always coexist simultaneously. Focusing on the individual effect of a single pollutant or meteorological factor may overlook the health effect of the overall mix. Effective methods to address this bias are required [6].

The influence of air pollution and meteorological factors leading to asthma exacerbation is also age-dependent. Previous studies have illustrated that asthmatic children are vulnerable to the adverse effects of air pollution because their lungs are still developing, their metabolic capacity is immature, and they have greater outdoor activity [25,26]. Older people with asthma are also susceptible to the adverse effects of air pollution [25]. Asthma exacerbations occur more often and are more severe in young boys than in girls, while active asthma is more prevalent among adult women than men [6,27]. Thus, the interplay between environment factors and individual characteristics leads to a particular phenotype. 

One strategy for reducing outdoor pollution-related asthma exacerbation is an early alert when an indicated environmental factor is predicted to be harmful. Here, we attempt to provide a comprehensive report regarding the influence of air pollution and meteorological factors on asthma AE for patients of different ages and sexes.

## 2. Materials and Methods 

### 2.1. Data Source

The study was approved by the Institutional Review Board of Chang Gung Memorial Hospital (201700710B0C501). The Ethics Committee and Institutional Review Board of Chang Gung Memorial Hospital waived the need for informed consent. The information obtained from each computerized claim form included age, sex, medical care institutions, and diagnosis of the patient. Both personal and medical care institutions’ identities in the database were omitted in compliance with the Personal Electronic Data Protection Law in Taiwan. The National Health Insurance Research Database was used as the data source for this study. Taiwan’s National Health Insurance program provides healthcare for 99% of the population [28]. A detailed description of Taiwan’s National Health Insurance Research Database (NHIRD) sample and procedures has been reported [29]. Briefly, this study used the claims data of 1 million subjects randomly selected from 23 million insurants registered from 2005 and traced to 2013 (approximately 5% of the total Taiwanese population). All methods were completed in accordance with relevant guidelines and regulations [30]. 

### 2.2. Patients and Outcome

The primary outcome measured was emergency room (ER) visits for asthma AE. Data were collected on all diagnoses for all patients from 1 January 2005 to 31 December 2013 in Taiwan. Individuals who were included had a diagnosis of asthma (International Classification of Diseases, Ninth Revision, Clinical Modification code 493.xx) as a principal or secondary condition resulting from an ER visit, as previously reported [31]. The study population was further divided into subgroups according to sex and age. 

### 2.3. Monitoring of Outdoor Air Pollution and Climate Factors 

**Figure 1:**
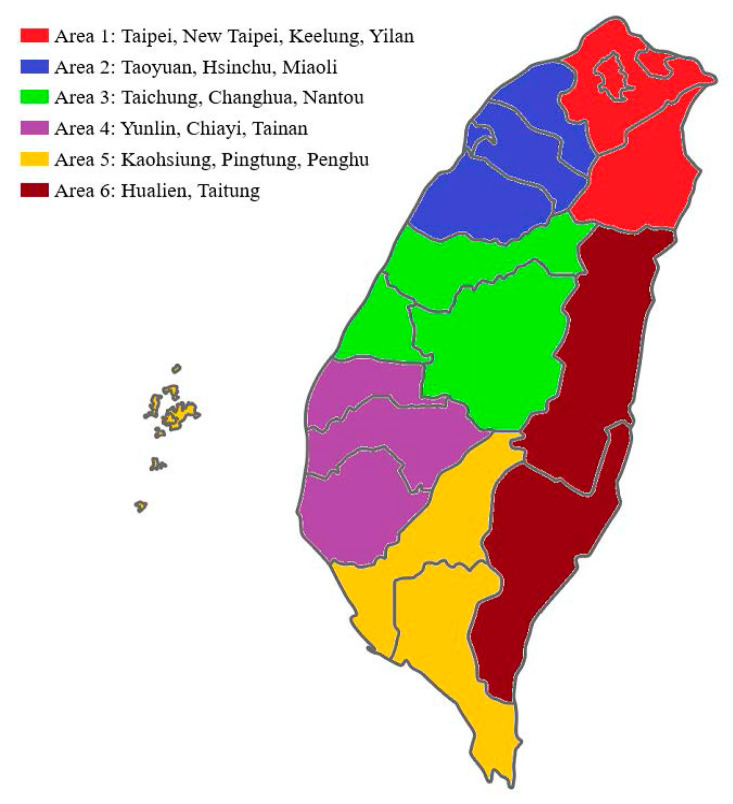
Geographic areas in Taiwan. Six geographic areas are grouped according to the Taiwan National Statistics of Regional Standard Classification (area 1: Taipei-New Taipei City-Keelung-Yilan; area 2: Taoyuan-Hsinchu-Miaoli; area 3: Taichung-Changhua-Nan-Tou county; area 4: Yunlin-Jiayi-Tainan; area 5: Kaohsiung- Pingdong; area 6: Hualia-Taidong).

Complete monitoring data for the air pollutants included SO_2_, NO_2_, O_3_, CO, PM_2.5_, and PM_10_. Meteorological factors, including relative humidity, rainfall, and daily average temperature, were also collected. For comparison by location, Taiwan was grouped into six geographic areas according to the Taiwan National Statistics of Regional Standard Classification (area 1: Taipei-New Taipei City-Keelung-Yilan); area 2: Taoyuan-Hsinchu-Miaoli; area 3: Taichung-Changhua-Nan-Tou county; area 4: Yunlin-Jiayi-Tainan; area 5: Kaohsiung- Pingdong; and area 6: Hualia-Taidong); (Figure 1) [29]. In Taiwan, there are 74 stations; in area 1 (an area of 4600.72 square kilometers), 21 stations; in area 2 (4468.79 square kilometers), 12 stations; in area 3 (7395.71 square kilometers), 12 stations; in area 4 (5386.11 square kilometers), 11 stations; in area 5 (5227.45 square kilometers), 15 stations; and in area 6 (8143.82 square kilometers), 3 stations. The air pollutant concentrations were obtained from the results gathered via total or local nearby geographic monitoring stations from the Environmental Protection Agency of the Taiwanese government as indicated. 

The 24-h average of air pollutants and meteorological factors were used to investigate the associations. The 48-h moving averages (including the concurrent day in which the ER visit occurred) of air pollution levels were most related to the daily ER visits for asthma. Air pollution generally had a 48-h lag effect [32]. Therefore, we used the 48-h moving averages of air pollutant concentrations and meteorological factors for the regression model. The odds ratios were expressed for each one unit increase in the concentration of air pollutants and meteorological factors, except for CO. Since a CO unit does not change by more than 1 ppm, we use 0.1 ppm as one unit of CO when analyzing the relationship between the CO level and ER visits for asthma AE.

### 2.4. Statistical Analysis

Data were presented as mean ± standard deviations (SD). Pearson’s correlation analysis was used to establish correlation coefficient (r) between each air pollutant and the weather factors. Multivariate binary logistic regression analysis by considering all factors (air pollution and meteorological factors) was used to explain how the degree of variance in the ER visits can be explained by a given set of air pollutants after adjusting for wind speed. *P* values were calculated to determine the significance of the regression relationships. An ANOVA test was used for multiple comparisons of different areas. *p* < 0.05 was considered statistically significant. All air pollutants and meteorological variables were modelled at the same time. Data analysis was performed using the SPSS 22.0 for Windows software package (SPSS, Chicago, IL, USA).

A case-crossover design has the advantage of controlling for potential confounding factors caused by fixing individual characteristics, such as sex, age, as well as underlying conditions. In this study, a bi-directional case-crossover design was used to control for time trends through the use of information regarding subjects both before and after the event [33]. For the case-crossover studies, data were analyzed by standard case-control methods. Exposures close in time to the event (case day) were contrasted with exposure at an indicated time when an event did not occur (control day). In the present study, each ER visit for asthma AE was defined as a case day. In the bi-directional control samplings, the same weekdays 1, 2 or 3 weeks before and after an ER visit for asthma were defined as the control days. For the same person, we compared outdoor pollution/meteorological factor exposure on the case day with air pollution exposure on the control days. Outdoor pollution/meteorological data were obtained from nearby geographic monitoring stations of the Environmental Protection Agency, based on each individual’s residential location. The same weekdays as the control periods were chosen to avoid a day-of-the-week effect and possible five-day lag effect [34]. Since individual-level covariates remained constant when comparing case days versus control days, they could be ignored and were not considered to be confounders. The association between asthma ER visits and outdoor pollution/meteorological factors was measured with an odds ratio using conditional logistic regression by STATA (StataCorp, College Station, TX, USA).

## 3. Results

### 3.1. Descriptive Statistics of the Number of ER Visits, Air Pollution Levels, and Meteorological Measures

**Figure 2:**
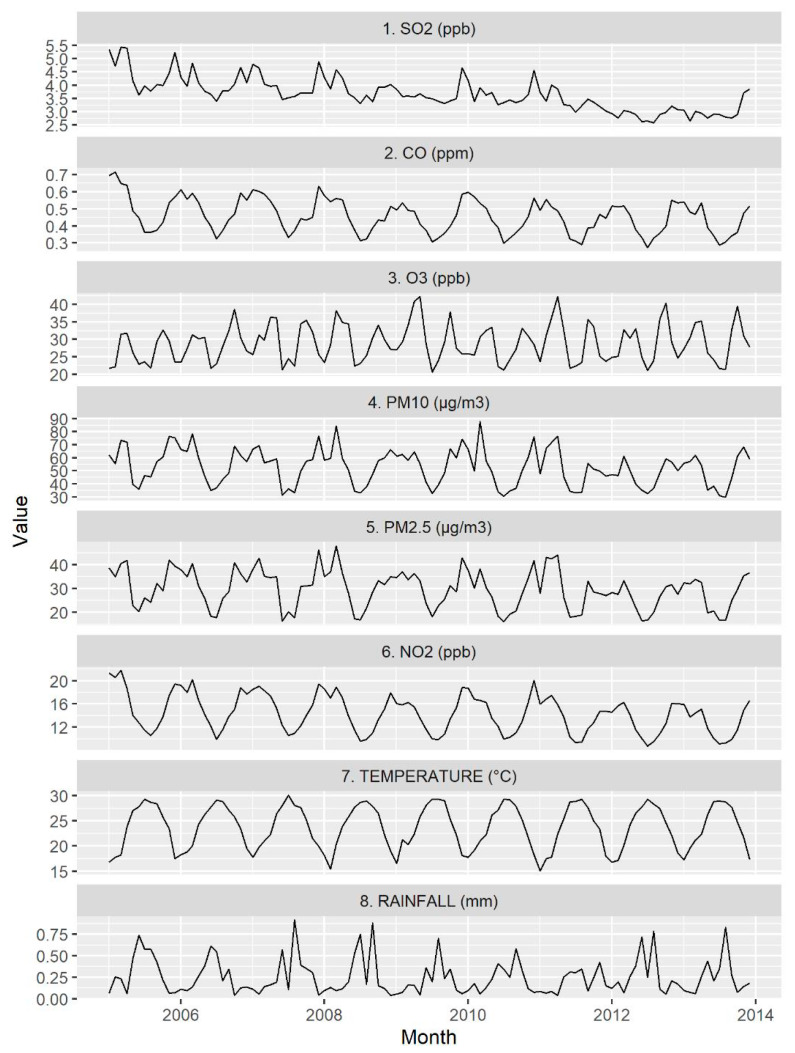
The timeframes for 24-h average of pollutants and meteorological factors from 2005 to 2013 in Taiwan.

Table 1 shows the total number of ER visits due to acute asthma in each month from 2005–2013. There were a total of 3287 days during this period. The baseline daily ER visits for asthma attack rate was 7.7 ± 4.0 per 1 million persons for the entire observation period. The 24-h PM_2.5_ average levels ranged from 8.1 to 97.1 μg/m^3^ (mean 29.8 ± 12.6 μg/m^3^). The lowest and highest value for a 24-h PM_10_ average level was 17.9 and 370.7 μg/m^3^, respectively (mean 53.1 ± 22.2 μg/m^3^). The timeframes for the 24-h average of pollutants and meteorological factors are shown in Figure 2. 

Of the 998,625 persons enrolled from 1 January 2005, there were 72,649 asthmatic patients with a prevalence rate of 7.27% (7.63% for men and 6.92% for women); (Supplementary Table S1). The prevalence rate of asthma for the pediatric group, adult group, and older age group was 13.51%, 3.94%, and 15.56%, respectively. The study population was randomly selected from 2005 and traced to 2013 without adding new cases. As these children grew up, the young adult group showed the most accumulative number of asthmatic patients since 2010 (Supplementary Table S1). Regarding ER visits for asthma AE, the pediatric group had the highest ER visit rate (40.1%) in 2005 among the various age groups (Supplementary Table S2). The cumulative number of ER visits for children decreased, and the young adult group showed the most cumulative number of ER visits for asthma attacks since 2010.

### 3.2. The High Correlation between the Level of Each Outdoor Air Pollutant and Meteorological Factors

Table 2 demonstrates the correlation of daily values over the entire period for the air pollutants and weather variables. The following pollutants: PM_2.5_, PM_10_, SO_2_, CO, and NO_2,_ had a strong positive correlation together and were negatively correlated with temperature, rainfall, and relative humidity. It is reasonable for a high degree of correlation to occur with PM_10_ and PM_2.5_. Outdoor air NO_2_ and CO were also noted to be highly correlated. Our results showed a decrease in outdoor air pollution with rainfall and more severe air pollution during the cold season. 

### 3.3. Profound Differences in Outdoor Air Pollutants among Various Geographic Areas

We attempted to compare the air pollution in various geographic areas. Taiwan is grouped into six geographic areas. An ANOVA analysis revealed substantial differences in each air pollutant among the various geographic areas in Taiwan (Supplementary Table S3). After plotting the outdoor air pollutants and meteorological factors by geographic area into heatmaps (Supplementary Figure S1), CO and NO_2_ levels were higher in areas 1, 2, and 3 than in other areas (Supplementary Figure S1A,B), while PM_2.5_ and PM_10_ levels were higher in areas 4 and 5 than in the others (Supplementary Figure S1C,D). Area 5 had the highest level of O_3_ and SO_2_ (Supplementary Figure S1E,F). As there were great variations in outdoor air pollutants among the various geographic areas, a more precise strategy to analyze the methods by which outdoor air pollutants/meteorological factors influence each asthma ER visit is required. 

**Figure 3:**
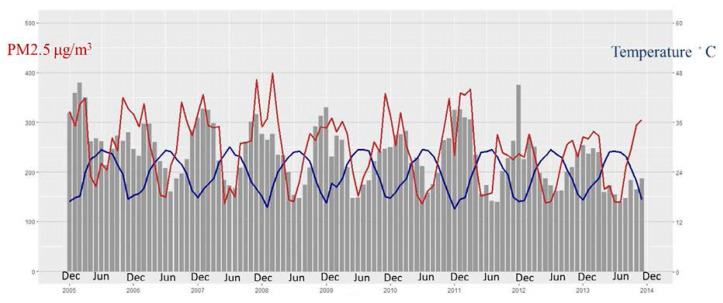
The time-series plot indicates the dynamic change in the national monthly number of visits to the ER for asthma (gray column), mean value of fine particulate matter (PM_2.5_) (red curve), and mean value of temperature (blue curve) across all locations. The left Y axis is the concentration of PM_2.5_ and the right Y axis is the degree of temperature.

When we combined the monthly mean values of PM_2.5_, mean temperature, and ER visits for asthma attacks, we found the number of ER visits for asthma AE to be positively correlated with the PM_2.5_ value (Figure 3). However, there were more ER visits due to asthma in the winter and spring, and fewer during summer and early autumn. Thus, it would be an inadequate analysis if only a single air pollutant is considered during asthma ER visits. As shown in Table 2, there was a close interplay between each air pollutant and meteorological factor. Therefore, the contribution of each air pollutant and meteorological factor to an asthma ER visit must be further clarified.

### 3.4. Relationship of Air Pollution/Meteorological Factors with ER Visits for Asthma in a Case Cross-Over Study

The case-crossover design was applied to evaluate the relationship between air pollution/meteorological factors and daily ER visits for acute asthma attacks. Individuals with a verified date of an ER visit for an asthma attack between 1 January 2005, and 31 December 2013, were included. During the study period, there were 25,167 case days and 149,442 control days. For a case-crossover analysis, a total of 25,167 ER visits (7.7 ± 4.0 per day in 3287 days) for asthma AE and 149,442 control days were included. The sex ratio of male to female was 56.7 to 43.3, with an age range of 18 to 64-years (37.6%). Here the meteorological factors were considered to be variables rather than adjusted factors because meteorological factors were also important risk factors for asthma AE in the real world. Using six reference periods (7, 14 and 21 days before and after the case period), a 1 μg/m^3^ increase in the 48-h average of PM_2.5_ and a 1 °C increase in temperature were associated with asthma ER visits (odds ratio (OR) = 1.004 (95% CI 1.001–1.007) and 0.986 (95% CI 0.980–0.991) respectively) (Table 3). Older people may have more chronic obstructive pulmonary disease (COPD) and heart failure. Asthma and COPD have a number of similarities, and it can be difficult to distinguish between them; therefore, people older than 64-years-old were excluded from further study. As the study cases were divided according to age, to our surprise, SO_2_ seemed to be a protective factor for children with acute asthma attacks. Each 1 ppb increase in in the 48-h average of SO_2_ were associated with an odds ratio of (OR) = 0.946 (95% CI 0.905–0.989) and 0.934 (95% CI, 0.879–0.991), respectively, for male and female children (Table 4). Each 1 ppb increase in the 48-h averages of O_3_ was associated with an asthma ER visit for female children (OR = 1.007 (95% CI, 1.000–1.013). A temperature increase was a protective factor for asthma ER visits, with a 1 °C increase in temperature associated with an OR = 0.982 (95% CI 0.970–0.994) and 0.979 (95% CI, 0.963–0.995) for male and female children, respectively. Each 1 °C increase in temperature was associated with an OR = 0.984 (95% CI 0.970–0.998) and 0.986 (95% CI, 0.973–0.999) for male and female young adults, respectively. We also analyzed the effect of air pollutants on asthma AE without including meteorological factors (Supplementary Table S4). If temperature, humidity, and rainfall were not considered, air pollutants showed different impacts on asthma AE. For example, O_3_ and NO_2_ will show harmful effects for asthma AE in the group of 0–17 years old boys and girls, respectively. Thus, meteorological factors are important and should be considered simultaneously with air pollution.

Since younger children may suffer higher rates of respiratory illnesses, we further stratified the pediatric group and determined the effects of air pollution and meteorological factors on asthma AE. However, the diagnosis of asthma in children younger than 5 years is rather difficult and frequently uncertain, thus they were excluded from analysis. The major impact of outdoor air pollution and meteorological factors on pediatric asthma AE mainly affected the age group of 6 to 11-year-olds (Table 5). A 1 mm/day increase in the 24-h average of rainfall and 1 °C increase in temperature were associated with asthma ER visits (OR = 0.897 (95% CI 0.816–0.986)) and 0.972 (95% CI 0.949–0.995) respectively) for the age group of 6 to 11-year-old boys. A 1 ppb increase in the 48-h averages of NO_2_ were associated with asthma ER visits (OR = 1.054 (95% CI 1.007–1.102)) for the age group of 6 to 11-year-old girls. The protective effect of SO_2_ was only seen in female adolescents with a 1 ppb increase in the 48-h average of SO_2_ associated with an odds ratio (OR) = 0.831 (95% CI 0.691–0.999).

## 4. Discussion

We conducted a comprehensive study to examine the influence of air pollution and meteorological factors by a bi-directional case-crossover analysis. We identified that temperature is an important protective factor for asthma AE both in asthmatic children and adults. Children, especially younger females, are more susceptible to asthma AE due to the effects of outdoor air pollution than adults.

The adverse impact of a single air pollutant on asthma has been confirmed [10,11,12,13,14,25,35]. Besides air pollution, meteorological factors such as temperature, atmospheric pressure and humidity also contributed to acute asthma attacks [20,22,23,24]. In the real world, the dynamic changes in air pollutants and meteorological factors always coexist simultaneously. The important meteorological factors will be ignored during environmental studies on asthma if only a single air pollutant is considered. Taiwan’s air quality problems are determined by multiple factors, such as topography, polluting industries concentrations, and motorcycle traffic compounds [36,37]. Fugitive dust at riverbanks during the low-flow season of winter, northeastern winds, and cross-border pollution from China also exacerbate the air pollution problem during winter [38,39]. Other studies have investigated the relationship between ER visits and PM_2.5_, showing that an increase in PM_2.5_ leads to more asthma ER visits in the warm season than in the cold season [35]. However, a high correlation between each outdoor air pollutant level and the meteorological factors was found in our study (Table 2). This observation was also supported by another study from Taiwan [40]. These inconsistent results varied by ethnicity and region. Respiratory tract infection was one of the important causes for asthma exacerbations and it is far more common in the cold season. Thus, the role of airway infection in the increase in asthma AE related to low temperature must be further clarified [5]. In addition to temperature, most air pollutant concentrations were negatively correlated with rainfall and humidity (Table 2), suggesting that air pollution is partially removed by rainfall.

There was a great variation in outdoor air pollutants among urban and suburban areas in Taiwan. Data from a nation-wide study cannot actually reflect the effects of outdoor air pollutants/meteorological factors on asthma ER visits. In the present study, we investigated the relationship between air pollution/meteorological factors and asthma ER visits by using a case-crossover study. The advantage of a case-crossover study is that the case serves as oneself referent to the control potential confounding factors when measuring the transient effects of intermittent exposure [41]. Compared with a previous report in Taiwan [42], the influence of air pollutants seems to be overestimated when using a population-based study.

It has been noted that the effects of air pollution on respiratory health is significantly modified by age and sex, although the results are inconsistent [43,44,45,46]. In general, studies in adults have shown that air pollutants had stronger effects on women. Studies in children also suggest stronger effects among girls in later life [43]. Our data also demonstrated a stronger association between pollutants (O_3_ and NO_2_) and asthma AE in girls with specific age groups. Whether these modifications are related to sociological, physiological, or both remain uncertain. Furthermore, we found that lower temperature and less rainfall are associated with acute asthma attacks among 6–11-year-old boys. Our work illustrated that in addition to air pollution, meteorological factors also have effects on the sexes of individuals.

The effect of SO_2_ on asthma has shown a high degree of heterogeneity in the literature [47]. Although the majority of reports have demonstrated the adverse effects of SO_2_ on asthma exacerbations, in our results, SO_2_ levels seemed to be associated with a decrease in asthma ER visits in children (Table 4). Girls aged 12 to 17-years-old were most affected (Table 5). A study reported by Villeneuve et al. also showed the protective effects of SO_2_ in certain seasons and age groups [48]. Other effect modifiers, which were not available in this study, could have influenced these results.

PM is a mixture of many substances. This contributes to the inconsistent associations between asthma prevalence and the exposure to outdoor PM reported in various areas [47]. Household/indoor PM is highly correlated with outdoor PM (even worse than outdoor PM under certain conditions) [49]. As people may spend more time indoors, indoor air pollutants may have a greater impact on their respiratory health. In cold weather, people may tend to remain indoors with closed windows and doors. Huang, C, et al showed that, in winter, indoor CO_2_ concentrations were significantly associated with the increased odds of childhood asthma in Shanghai [49]. The effect of indoor pollutants on asthma AE is an interesting and important issue that is well worth investigating. Other factors that have an influence on asthma AE but are not considered in this work include allergen exposure. Seasonal pollen allergens may be a component of PM that may contribute to asthma AE [50]. However, in Taiwan, more than 80% of atopic individuals are sensitized to house dust mites, an annual aeroallergen, in contrast to only 3% to grass pollen [51]. Thus, seasonal allergen is not considered in this study.

Our study has other limitations. First, this case-crossover design does not provide an estimate of the increase in asthma ER visits that are associated with long-term exposure to air pollution. We also did not analyze the effects of peak values of air pollutants on asthma AE here. Although there are limited reports showing that brief exposures to high concentrations of SO_2_ do not cause serious symptoms in asthmatic patients [52], we still need further studies to validate the effect of peak value of other pollutants on asthma. Second, because this study used exposure levels’ nearby residential locations rather than exact home and school addresses or places of employment, it may not reflect the actual exposure. Third, this study demonstrated general behavior for medical visits but not for all special conditions. For example, people may delay ER visits during heavy rain. People may also stay indoors or wear a mask when they receive air pollution warnings. All these additional behaviors may change the impact of air pollution and meteorological factors. Besides, as the accuracy of the asthma diagnosis was based on administrative data, we were not able to analyze the heterogenous characteristics from population-based data due to the limitations posed by personal data availability. 

## 5. Conclusions

The precise collaborative influences of several combined air pollutants and meteorological factors are important and significant. Meteorological factors are important modulators for asthma AE both in asthmatic children and adults. When studying the effects of air pollution on asthma AE, meteorological factors should be considered.

## Figures and Tables

**Table 1:** Descriptive statistics of air pollution and meteorological measures (total 3287 days).

Characters	Mean ± SD	Min	1st Qu.	Median	3rd Qu.	Max
Number of ER visit (per day)	7.7 ± 4.0	0	5	7	10	33
Number of Hospitalization	3.4 ± 2.1	0	2	3	5	14
Air pollutants concentrations						
SO_2_ (ppb)	3.7 ± 1.0	1.5	3.1	3.6	4.1	9.8
CO (ppm)	0.5 ± 0.1	0.2	0.4	0.4	0.5	1.2
O_3_ (ppb)	28.9 ± 8.5	9.6	22.6	27.6	33.9	67.6
PM_10_ (μg/m^3^)	53.1 ± 22.2	17.9	36.8	49.2	65.0	370.7
PM_2.5_ (μg/m^3^)	29.8 ± 12.6	8.1	20.0	27.5	37.3	97.1
NO_2_ (ppb)	14.4 ± 4.1	3.3	11.0	14.0	16.9	33.9
Meteorological measures						
Temperature (°C)	23.8 ± 4.7	10.1	20.0	24.6	28.0	31.3
Rainfall (mm/day)	0.3 ± 0.6	0	0.0	0.1	0.2	8.5

Air pollutants concentrations and meteorological measures are 24-h average. Abbreviation: Qu.: quarter.

**Table 2:** Pearson correlation coefficients for nation-wide daily weather and air pollution variables in Taiwan from Jan 2005 to Dec 2013.

Factors	SO_2_	CO	O_3_	PM_10_	PM_2.5_	NO_2_	Temperature	Rainfall	Relative Humidity
SO_2_	1								
CO	0.6	1							
O_3_	0.1	0.1	1						
PM_10_	0.6	0.6	0.5	1					
PM_2.5_	0.7	0.7	0.5	0.9	1				
NO_2_	0.7	0.9	0.2	0.6	0.7	1			
Temperature	−0.3	−0.6	−0.1	−0.4	−0.4	−0.7	1		
Rainfall	−0.3	−0.2	−0.2	−0.3	−0.3	−0.3	0.2	1	
Relative Humidity	−0.5	−0.0	−0.4	−0.4	−0.4	−0.1	0.1	0.5	1

A 24-h average of air pollutants and meteorological factors was used to investigate their association.

**Table 3:** Relationship of air pollution/meteorological factors with ER visits for asthma in a case cross-over study without stratification by age.

Factors	OR	95% CI	*p*-Value
All			
SO_2_	0.954	0.934–0.974	<0.001
CO	1.002	0.976–1.028	0.898
O_3_	1.002	1.000–1.004	0.126
PM_10_	0.999	0.998–1.001	0.235
PM_2.5_	1.004	1.001–1.007	0.017
NO_2_	0.999	0.992–1.007	0.788
Temperature	0.986	0.980–0.991	<0.001
Rainfall	0.991	0.972–1.011	0.390
Relative humidity	0.998	0.996–1.001	0.197

The effect for SO_2_ is analyzed for 1ppb, for CO is 0.1 ppm, for O_3_ is 1 ppb, for PM_2.5_ and PM_10_ is 1 μg/m^3^, for NO_2_ is 1 ppb, for temperature is 1 °C, for rainfall is mm/day, for relative humidity is 1%.

**Table 4:** Relationship of air pollution/meteorological factors with ER visits for children and adults with asthma in a case cross-over study

Factors	Male			Female		
	OR	95% CI	*p*-Value	OR	95% CI	*p*-Value
0–17-year-olds						
SO_2_	0.946	0.905–0.989	0.013	0.934	0.879–0.991	0.024
CO	0.987	0.934–1.042	0.628	0.944	0.875–1.019	0.138
O_3_	1.004	0.999–1.009	0.097	1.007	1.000–1.013	0.041
PM_10_	1.001	0.999–1.003	0.362	1.001	0.997–1.004	0.753
PM_2.5_	0.999	0.994–1.005	0.780	1.004	0.996–1.012	0.343
NO_2_	1.005	0.989–1.021	0.549	1.019	0.997–1.041	0.085
Temperature	0.982	0.970–0.994	0.003	0.979	0.963–0.995	0.012
Rainfall	0.963	0.922–1.006	0.089	0.972	0.914–1.034	0.375
Relative humidity	1.002	0.997–1.008	0.437	0.999	0.992–1.007	0.860
18–64-year-olds						
SO_2_	0.963	0.915–1.012	0.139	0.968	0.923–1.016	0.191
CO	1.027	0.967–1.092	0.384	1.035	0.976–1.098	0.252
O_3_	1.001	0.996–1.006	0.656	1.001	0.996–1.006	0.713
PM_10_	0.999	0.995–1.002	0.418	0.998	0.994–1.002	0.281
PM_2.5_	1.004	0.997–1.011	0.254	1.003	0.996–1.010	0.401
NO_2_	0.987	0.969–1.004	0.140	0.99	0.974–1.007	0.267
Temperature	0.984	0.970–0.998	0.021	0.986	0.973–0.999	0.032
Rainfall	1.019	0.978–1.062	0.372	1.028	0.984–1.073	0.221
Relative humidity	0.999	0.993–1.005	0.757	0.996	0.990–1.002	0.149

The effect for SO_2_ is analyzed for 1 ppb, for CO is 0.1 ppm, for O_3_ is 1 ppb, for PM_2.5_ and PM_10_ is 1 μg/m^3^, for NO_2_ is 1 ppb, for temperature is 1 °C, for rainfall is mm/day, for relative humidity is 1%.

**Table 5:** Relationship of air pollution/meteorological factors with ER visits for pediatric asthma further stratified by age

Factors	Male			Female		
	OR	95% CI	*p*-Value	OR	95% CI	*p*-Value
6–11-year-olds						
SO_2_	0.929	0.857–1.008	0.076	0.952	0.843–1.076	0.432
CO	0.989	0.892–1.096	0.833	0.873	0.739–1.031	0.109
O_3_	1.007	0.998–1.017	0.110	1.014	1.000–1.029	0.056
PM_10_	1.004	1.000–1.009	0.077	1.001	0.992–1.010	0.823
PM_2.5_	0.997	0.986–1.007	0.541	0.995	0.977–1.014	0.591
NO_2_	1.012	0.983–1.042	0.421	1.054	1.007–1.102	0.023
Temperature	0.972	0.949–0.995	0.018	0.969	0.934–1.005	0.086
Rainfall	0.897	0.816–0.986	0.025	1.019	0.887–1.171	0.790
Relative humidity	1.009	0.998–1.021	0.106	0.995	0.978–1.012	0.559
12–17-year-olds						
SO_2_	0.983	0.854–1.131	0.806	0.831	0.691–0.999	0.049
CO	0.858	0.725–1.017	0.077	0.876	0.697–1.101	0.256
O_3_	1.011	0.997–1.005	0.123	0.997	0.978–1.015	0.719
PM_10_	0.998	0.990–0.123	0.550	1.004	0.997–1.012	0.270
PM_2.5_	1.006	0.988–1.024	0.531	1.006	0.982–1.030	0.624
NO_2_	1.030	0.983–1.080	0.215	1.049	0.983–1.120	0.145
Temperature	0.981	0.945–1.019	0.322	0.992	0.943–1.044	0.769
Rainfall	0.961	0.836–1.104	0.571	1.088	0.940–1.258	0.258
Relative humidity	1.007	0.990–1.0.24	0.432	1.000	0.979–1.022	0.997

The effect for SO_2_ is analyzed for 1 ppb, for CO is 0.1 ppm, for O_3_ is 1 ppb, for PM_2.5_ and PM_10_ is 1 μg/m^3^, for NO_2_ is 1 ppb, for temperature is 1 °C, for rainfall is mm/day, for relative humidity is 1%.

## Supplementary Materials

The following are available online at https://www.mdpi.com/1660-4601/17/11/4010/s1, Figure S1: Daily mean (A) carbon monoxide (CO), (B) nitrogen dioxide (NO_2_), (C) fine particulate matter (PM _2.5_), (D) PM_10_, (E) ozone (O_3_), (F) and sulfur dioxide (SO_2_) concentrations were calculated and plotted by geographic area., Table S1: The accumulative number of asthmatic patients from 2005 to 2013 in one million population, Table S2: The accumulative number of ER visit for asthma acute attack from 2005 to 2013 in one million population, Table S3: Analyze the difference of air pollutant among different geographic areas in Taiwan ANOVA test, Table S4: The relationship of air pollution to ER visits for asthma by case cross-over study (without meteorological factor).
