# Supplementary material for: A Multifactorial Evaluation of the Effects of Air Pollution and Meteorological Factors on Asthma Exacerbation
Source: Int J Environ Res Public Health. 2020 Jun 4;17(11):4010. doi: 10.3390/ijerph17114010 (PMC7313451; doi:10.3390/ijerph17114010)
Supplement: Supplementary file 1 [file ijerph-17-04010-s001.pdf]

Supplementary Table S1. The accumulative number of asthmatic patients from 2005 to 2013 in one million population.

Year		2005	2006	2007	2008	2009	2010	2011	2012	2013
Sex										
	Male	37,764/495,116 (7.63 %)	40,777	43,684	45,992	48,043	49,665	51,219	52,388	53,184
	Female	34,885/503,509 (6.92 %)	37,787	40,633	43,244	45,444	47,385	49,211	50,647	51,552
Age										
	0–17	30,580/226,294 (13.51 %)	32,713	34,561	35,599	35,918	35,502	34,344	32,554	30,159
	18–64	26,525/672,457 (3.94 %)	28,562	30,593	32,808	35,182	37,676	40,564	43,300	45,853
	65-	15,544/99,875 (15.56 %)	17,289	19,163	20,829	22,387	23,872	25,522	27,181	28,724
Total		72,649/998,625 (7.27 %)	78,564	84,317	89,236	93,487	97,050	100,430	103,035	104,736

Supplementary Table S2. The accumulative number of ER visit for asthma acute attack from 2005 to 2013 in one million population.

Year		2005	2006	2007	2008	2009	2010	2011	2012	2013	Total
Sex											
	Male	2014 (57.9%)	1703 (60.5%)	1821 (58.9%)	1565 (56.4%)	1501 (56.2%)	1617 (57.6%)	1509 (53.8%)	1427 (53.8%)	1226 (53.7%)	14,383
	Female	1464 (42.1%)	1114 (39.5%)	1270 (41.1%)	1211 (43.6%)	1170 (43.8%)	1189 (42.4%)	1294 (46.2%)	1225 (46.2%)	1059 (46.3%)	10,996
Age											
	0–17	1393 (40.1%)	1150 (40.8%)	1281 (41.4%)	1106 (39.8%)	974 (36.5%)	958 (34.1%)	745 (26.6%)	677 (25.5%)	449 (19.6%)	8733
	18–64	1231 (35.4%)	1031 (36.6%)	1055 (34.1%)	937 (33.8%)	964 (36.1%)	1023 (36.5%)	1183 (42.2%)	1092 (41.2%)	1032 (45.2%)	9548
	65-	854 (24.6%)	636 (22.6%)	755 (24.4%)	733 (26.4%)	733 (27.4%)	825 (29.4%)	875 (31.2%)	883 (33.3%)	804 (35.2%)	7098
Total		3478	2817	3091	2776	2671	2806	2803	2652	2285	25,379

Supplementary Table S3. Analyze the difference of air pollutant among different geographic areas in Taiwan ANONA test.

	Df	Sum Sq	Mean Sq	F-Value	*p*-Value
SO2					
between groups	5	657.60	131.52	214.10	<0.001
within groups	642	394.30	0.61		
CO					
between groups	5	1.58	0.32	26.57	<0.001
within groups	642	7.66	0.01		
O3					
between groups	5	4238.00	847.60	23.26	<0.001
within groups	642	23,391.00	36.40		
PM10					
between groups	5	119,165.00	23,833.00	91.77	<0.001
within groups	642	166,724.00	260.00		
PM2.5					
between groups	5	42,355.00	8471.00	97.57	<0.001
within groups	642	55,302.00	87.00		
NO2					
between groups	5	7179.00	1435.90	103.70	<0.001
within groups	642	8887.00	13.80		

24-h average of air pollutants and meteorological factors were analyzed.

Supplementary Table S4. The relationship of air pollution to ED visits for asthma by case cross-over study (without meteorologic factor).

Factors	Male			Female		
	OR	95% CI	*p*-value	OR	95% CI	*p*-value
0–17-year-old						
SO_2	0.932	0.879–0.988	0.018	0.948	0.871–1.031	0.214
CO	0.964	0.893–1.041	0.354	0.846	0.753–0.951	0.005
O_3	1.007	1.000–1.014	0.043	1.008	0.998–1.018	0.118
PM10	1.001	0.998–1.005	0.476	1.003	0.998–1.009	0.265
PM2.5	0.999	0.990–1.007	0.742	0.997	0.984–1.010	0.639
NO_2	1.019	0.996–1.042	0.108	1.057	1.023–1.093	0.001
18–64-year-old						
SO_2	0.932	0.878–0.988	0.018	1.006	0.951–1.065	0.838
CO	0.993	0.922–1.069	0.853	0.999	0.927–1.075	0.969
O_3	1.001	0.995–1.007	0.735	1.000	0.994–1.007	0.915
PM10	1.000	0.995–1.004	0.849	0.998	0.993–1.003	0.440
PM2.5	1.003	0.994–1.011	0.547	1.003	0.994–1.012	0.534
NO_2	0.998	0.976–1.020	0.844	0.994	0.973–1.016	0.579

The effect for SO_2 is analyzed for 1 ppb, for CO is 0.1 ppm, for O_3 is 1 ppb, for $PM_{2.5}$ and PM_{10} is 1 μg/m^3, for NO2 is 1 ppb, for temperature is 1 °C, for rainfall is mm/day, for relative humidity is 1%.

Supplementary figure 1

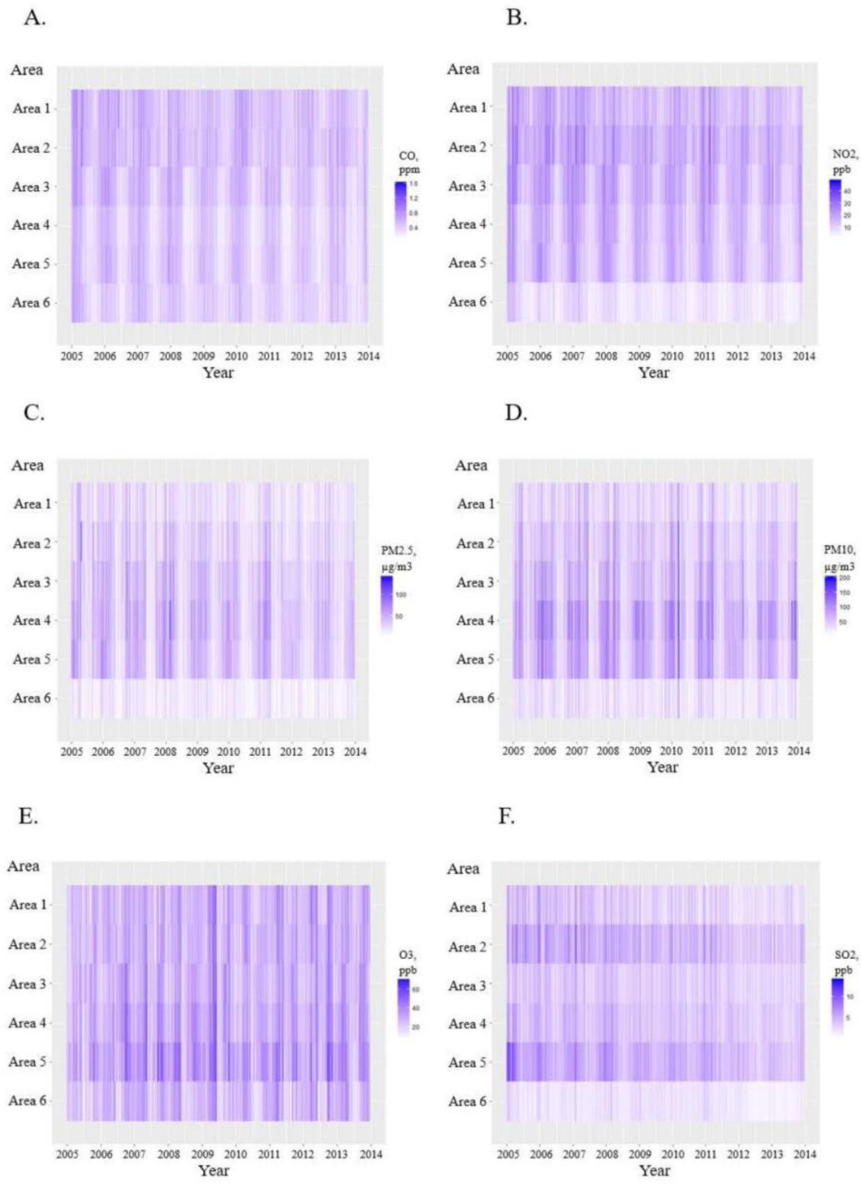

Figure S1. Daily mean (**A**) carbon monoxide (CO), (**B**) nitrogen dioxide (NO_2), (**C**) fine particulate matter (PM 2.5), (**D**) PM10, (**E**) ozone (O_3), (**F**) and sulfur dioxide (SO_2) concentrations were calculated and plotted by geographic area.
